# A radiolabeled drug tracing method to study neurotrophin-3 retention and distribution in the cochlea after nano-based local delivery

**DOI:** 10.1016/j.mex.2020.101078

**Published:** 2020-09-24

**Authors:** Patrick Lam, Niliksha Gunewardene, Yutian Ma, Frank Caruso, Trung Nguyen, Brianna Flynn, Andrew K. Wise, Rachael T. Richardson

**Affiliations:** a Bionics Institute, East Melbourne, Victoria 3002, Australia; b Department of Medical Bionics, University of Melbourne, Fitzroy, Victoria 3065, Australia; c ARC Centre of Excellence in Convergent Bio-Nano Science and Technology, and the Department of Chemical Engineering, University of Melbourne, Parkville, Victoria 3010, Australia; d University of Melbourne, Department of Otolaryngology, The Royal Victorian Eye and Ear Hospital, East Melbourne, VIC 3002, Australia

**Keywords:** Drug delivery, Nanoparticles, Neurotrophins, Hearing loss, Inner ear, Cochlea, Protein therapy

## Abstract

Hearing loss is the most common sensory deficit worldwide with no approved therapeutics for treatment. Local neurotrophin delivery into the cochlea has shown great potential in protecting and repairing the sensory cells important for hearing. However, delivery of these factors into the inner ear at therapeutic levels over a sustained period of time has remained a challenge restricting clinical translation. We have developed a method to test the pharmacokinetics of neurotrophin released from porous silica particles called ‘supraparticles’ that can provide sustained release of neurotrophins to the inner ear.

• This report describes a radiolabeling method to examine neurotrophin retention and distribution in the cochlea. The neurotrophin was labeled with a radioactive tracer (iodine 125: ^125^I) and delivered into the cochlea via the supraparticle system.
• Gamma counts reveal drug levels and clearance in the intact cochlea, as well as accumulation in off-target organs (safety test). Autoradiography analyses using film and emulsion permit quantification and visualization of drug distribution at the cellular level. The method has a detection limit of 0.8 pg of radiolabeled neurotrophin-3 in cochlear sections exposed to film.
• The tracer ^125^I with a half-life of 59.4 days can be used to label other drugs/substances with a tyrosine residue and therefore be broadly applicable for long-term pharmacokinetic studies in other systems.

## Graphical abstract

**Figure:**
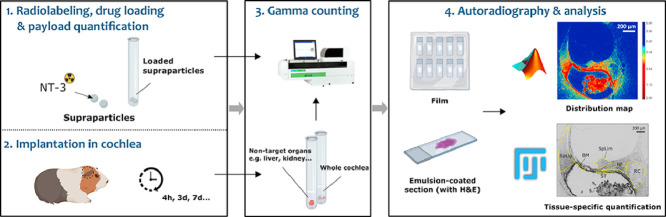

**Specifications table**

**Table:** 

**Subject Area**	Neuroscience
**More specific subject area**	*Drug delivery and Pharmacokinetics*
**Method name**	**Radiolabeled drug tracing method for cochlear neurotrophin pharmacokinetic study**
**Name and reference of original method**	Richardson, R.T., et al., *Tracing neurotrophin-3 diffusion and uptake in the guinea pig cochlea.* Hearing Research, 2004. **198**(1): p. 25–35. Richardson, R.T., et al., *A single dose of neurotrophin-3 to the cochlea surrounds spiral ganglion neurons and provides trophic support.* Hearing Research, 2005. **204**(1): p. 37–47. Richardson, R.T., et al., *Pharmacokinetics and tissue distribution of neurotrophin 3 after intracochlear delivery.* Journal of Controlled Release, 2019. **299**: p. 53–63.
**Resource availability**	*If applicable, include links to resources necessary to reproduce the method (*e.g. *data, software, hardware, reagent)* Optical density calibration https://imagej.nih.gov/ij/docs/examples/calibration/

## Method background

Hearing loss most commonly occurs as a result of damage to the sensory hair cells and auditory neurons in the cochlea. It is well-established that neurotrophins are a potential therapy for hearing loss due to their capacity to protect and regenerate auditory neurons. However, clinical translation of the therapy has been impeded by the lack of a clinically translatable drug delivery system that provides sustained neurotrophin release to the cochlea. We have developed a drug delivery system comprised of mesoporous silica nanoparticles that are formed into supraparticles [1]. Supraparticles allow neurotrophins to be loaded in large doses and released over time [1]. We have previously shown that supraparticle-released neurotrophins significantly improve auditory neuron survival in the cochlea, but the pharmacokinetics of the released neurotrophin remained to be determined [2].

The testing of drug delivery approaches for the inner ear requires a pharmacokinetic technique that permits evaluation of drug levels, distribution and clearance from the target tissue with both spatial and temporal resolution. Techniques such as serial perilymph sampling – the standard cochlear pharmacokinetic method - provides information on drug levels in cochlear fluids at set time points. However, the sampling method does not take into account drug bound to cochlear tissue unavailable for extraction and provides no information on the extent of drug uptake at the cellular level. Furthermore, fluid contamination from cerebrospinal fluid may also compromise the data especially if the sampling volumes are inaccurate. Other approaches such as quantitative MRI/micro CT imaging [3,4], electrophysiological measurements [5], electrochemical measurements [6], fluorescently-labeled drug conjugates or tracers [7], [8], [9] have also been tested to evaluate cochlear pharmacokinetics, but these techniques provided pharmacokinetic information over only short durations of time (1 h-3 days). Although the fluorescence-based technique allows visualization of drug uptake at cellular level, the use of relatively large fluorophores can alter the drug behavior thus limiting the utility of this approach in understanding drug pharmacokinetics [10]. A comparison of different techniques used for inner ear pharmacokinetics evaluation is provided in Supplementary Table 1.

We previously reported a relatively simple assay involving radiolabeled drug tracing to assess the cochlear pharmacokinetics of a bolus dose of neurotrophin-3 (NT-3) injection [11–13]. We have adapted this system to study the pharmacokinetics of NT-3 delivered into the cochlea via the supraparticle system. The neurotrophin is labeled with a radioactive tracer (iodine 125: ^125^I) loaded into the supraparticles and implanted into guinea pig cochleae. The method permits analysis of the amount of neurotrophin loaded into the supraparticles and its retention, clearance and distribution in the cochlea. Furthermore, it enables analysis of drug uptake in the target tissues, drug accumulation in other non-target tissues as a safety indication, and can be applied to long-term treatment studies, within the confines of ^125^I decay half-life of 59.4 days. Herein, we describe this radiolabeled drug tracing method in further detail.

## Method details

### Neurotrophin preparation and radiolabeling

Reconstituted human NT-3 (3–4.5 µg; PeproTech, New Jersey, USA) was radio-isotopically labeled with 0.25 mCi Na ^125^I (ICN Biomedicals Australasia) via the Chloramine-T method (ProSearch International) [14]. After the halogenation reaction, unbound radioiodine was removed from ^125^I labeled NT-3 (^125^I-NT-3) in a Biogel P6DG chromatography column. ^125^I-NT-3 was preserved in phosphate buffer containing 0.25% (w/v) BSA and 0.1% (w/v) sodium azide. The specific radioactivity of ^125^I-NT-3 used for this study was 46 and 46.7 µCi/µg, with 82 and 84% incorporation.

^125^I-NT-3 was then subject to purification and buffer exchange using an Amicon Ultra-2 3 K filtration unit. Prior to use, the filter was pre-coated in PBS containing 1% (w/v) BSA. After 20 min of pre-coating, the solution was centrifuged through the filter for 2 min and discarded. One milliliter of ^125^I-NT-3 was then added to the filter and centrifuged at 5500 × g for 10 min. The ultrafiltrate was discarded and the filter unit containing the concentrated ^125^I-NT-3 was replenished with artificial perilymph (125 mM NaCl, 3.5 mM KCl, 1.3 mM CaCl_2_, 25 mM NaHCO_3_, 1.2 mM MgCl_2_, 0.75 mM NaH_2_PO_4_, 5 mM glucose, pH 7.4). The filtration unit was then centrifuged at 5500 × g for 10 min and the final ^125^I-NT-3 solution collected. The volumes of the ultrafiltrates and concentrates were noted and their radioactivity measured using a Perkin Elmer WIZARD automatic gamma counter to calculate the final ^125^I-NT-3 concentration. The ^125^I-NT-3 was adjusted to 1 µg/mL prior to supraparticle loading.

### Neurotrophin loading and payload quantification

Supraparticles were sterilized in 100 µL of 80% (v/v) ethanol at room temperature for 4 h and washed 6 times with Milli-Q water and once with 0.1 M PBS prior to drug loading. A trace amount of radiolabeled NT-3 was mixed with native (unlabeled) NT-3 at a 1:1 volume ratio and a concentration ratio of 1:1000. For intracochlear surgeries, supraparticles were loaded in batches of 2 supraparticles in a solution containing 7.5 µL of NT-3 (stock 2 mg/mL) and ^125^I-NT-3 (stock 2.7 or 3.1 µg/mL) in a total volume of 15 µL. Immediately prior to implantation, the supraparticles were washed three times with Milli-Q water and gamma counts were taken leaving the supraparticles in Milli-Q water to determine drug loading. Radioactivity in post-loading NT-3 solution, washes, used pipette tips, and cannulas were then measured in order to determine the residual and lost amount of NT-3, and to characterize the loading efficiency. Based on the readings of pre/post-loading solutions, radiolabel incorporation rates, and the ^125^I-NT-3 to native NT-3 concentration ratio (1:1000), the total amount of NT-3 loaded to each supraparticle was calculated. The supraparticle loading capacity for both labeled and unlabeled NT-3 was characterized based on an averaged payload value.

### Supraparticle implantation

#### Animals

We used young adult pigmented guinea pigs of either sex (mean 550 g) in this study. All procedures were approved by the St. Vincent's Hospital Animal Research & Ethics Committee in accordance with the National Institutes of Health Guidelines for the Care and Use of Laboratory Animals and conformed to the Code of Practice of the National Health and Medical Research Council of Australia.

#### Intracochlear delivery

Prior to implantation, the guinea pigs were anesthetized via an intramuscular injection of 60 mg/kg ketamine (100 mg/mL; Parnell, Australia) and 4 mg/kg xylazine (20 mg/mL; Ilium, Australia), as well as a local injection of 0.5 mL/kg lignocaine hydrochloride (20 mg/mL; Troy Laboratories Pty Ltd., Australia) at the incision site. A post-auricular incision was performed to expose the auditory bulla on each side. A small hole was drilled in the bulla to expose the cochlear basal turn. A cochleostomy was performed at this site using a 0.8 mm diamond drill to create an opening (ca. 800 µm). Bony debris produced during the surgery was removed by gentle suction. For each cochlea, one NT-3 loaded supraparticle was transferred via a 21-gauge polyurethane catheter (Optiva, Medex Medical, UK) and placed into the scala tympani of the basal turn through the surgical opening. After supraparticle implantation, a silicone plug prepared in our laboratory was placed in the cochleostomy to seal the opening in the cochlea. The surgical incision site was held together by suturing two layers of muscles and stapling the top-layer skin at the site. Hartmann's solution (10 mL/kg; Sykes Vet International, Australia) and analgesic buprenorphine (50 mg/kg; Temgesic, Reckitt-Benckiser, UK) were injected subcutaneously on the day of surgery and the next day to assist with recovery and relieve pain. Antibiotic enrofloxacin (0.10 mg/kg; Baytril, Bayer, Germany) was given subcutaneously on the day of surgery and two days post-surgery to prevent infections.

#### Cochlea extraction

After treatment, the guinea pigs were intraperitoneally administered with a lethal dose of pentobarbitone sodium (150 mg/kg) (Lethabarb, Virbac, Australia). Transcardial perfusion and fixation was performed using 0.9% (w/v) saline (37 °C) containing 0.1% (w/v) heparin sodium and 0.025% (w/v) sodium nitrite followed by 4 °C 10% Neutral Buffered Formalin (10% NBF, Sigma, USA). The bulla was dissected out and perforated to create a small opening for cochlear inspection. The bulla was then carefully removed. Each treated cochlea, including the attached vestibular apparatus, was placed in 0.5 mL 10% NBF in a radioimmunoassay tube for gamma counting. The cochlear fluids and the implanted supraparticle remain in place and were included in the total count. Any unexpected fluid loss from the cochlea during the dissection process was captured into the same radioimmunoassay tube.

#### Whole-cochlea drug retention quantification

All the cochlear samples were measured for radioactivity within 24 h after the treatment. The percentage of NT-3 retention in each cochlea was estimated based on a ratio of the whole-cochlear radioactivity post-treatment to the radioactivity of NT-3 loaded supraparticles (adjustments in measurements were applied to account for the natural radioactive decay at the different time points of analysis). The total remaining amount of NT-3 in each cochlea was calculated using the corresponding pre-implantation supraparticle drug payload data as well as the NT-3 retention rates. This step is crucial in producing a snapshot of NT-3 retention level at a series of treatment time points.

### Histology

**Fig. 1:**
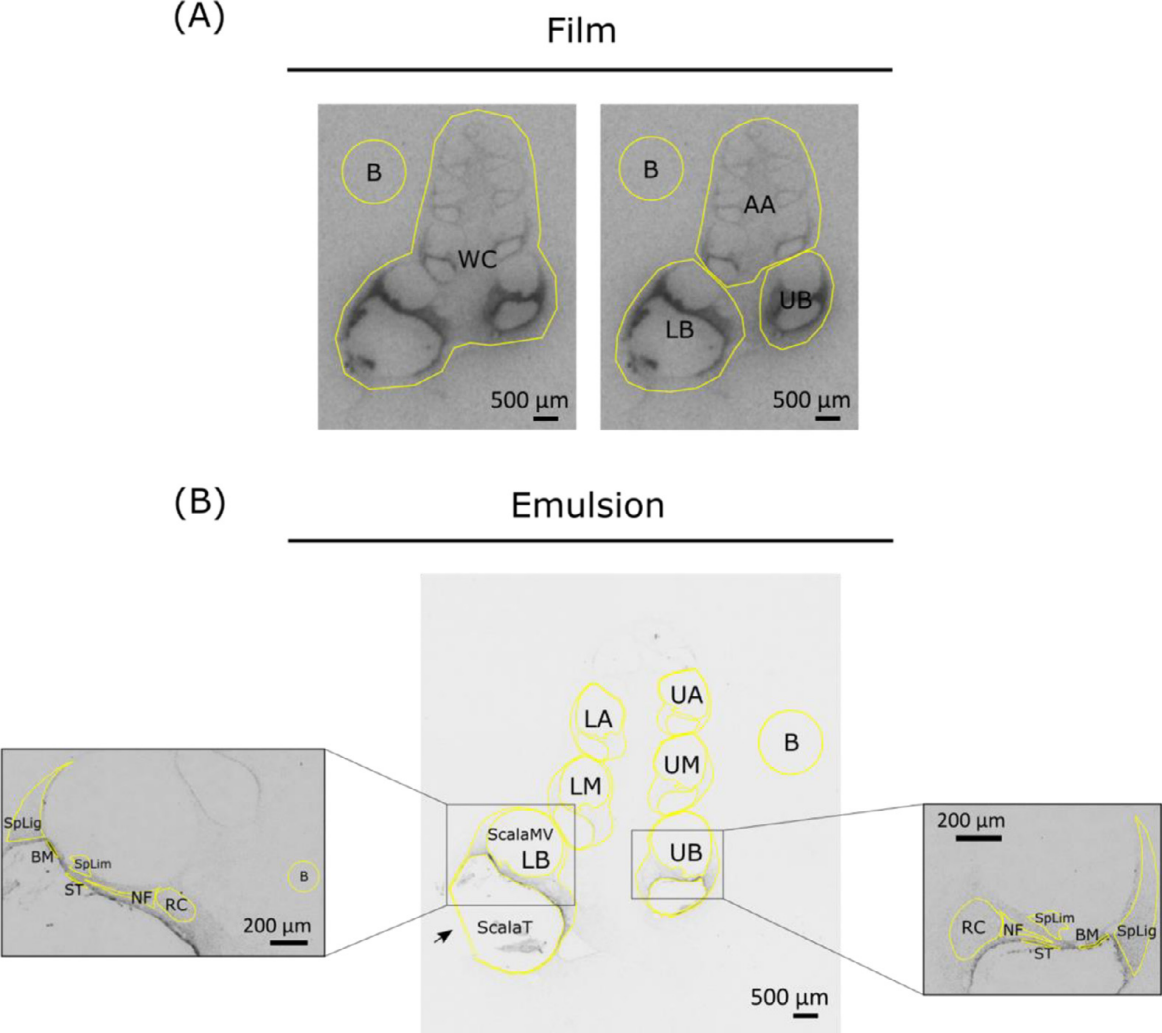
Regions of interest for film- and emulsion-based radiolabel signal quantification. (A) Four ROIs drawn to fit all selected film cochlear (mid-modiolar) sections using a polygon drawing tool in ImageJ. *B* = background; WC = whole cochlea; LB = lower basal turn; UB = upper basal turn; AA = all consecutive turns above basal turns. (B) Example of ROIs drawn for 6 cochlear turns and 6 types of tissue in the basal turns in each selected emulsion-coated mid-modiolar section using a freehand tool in ImageJ. Three fluid-filled chambers were excluded from the ROI of each cochlear turn. *B* = background; LB = lower basal turn; UB = upper basal turn; LM = lower middle turn; UM = upper middle turn; LA = lower apical turn; UA = upper apical turn; ScalaT = scala tympani; ScalaMV = scala media and scala vestibuli; BM = basilar membrane; SpLig = spiral ligament; SpLim = spiral limbus; RC = Rosenthal's canal; NF = nerve fibers; ST = mesothelial cell layer lining the scala tympani.

Following gamma measurements, cochlear samples with supraparticles *in situ* were prepared for cryo-sectioning. The vestibular apparatus was dissected apart from each cochlea. The cochleae were then decalcified in 10% ethylenediaminetetraacetic acid (EDTA) in PBS at room temperature over one to two weeks. Following decalcification, the cochleae were cryo-preserved in 15% (w/v) and then 30% (w/v) sucrose solution overnight at 4 °C. The next day, the cochleae were embedded in Tissue-Tek optimal cutting temperature (OCT) compound (Sakura, Japan), oriented, quick-frozen in liquid nitrogen or a dry ice/ethanol slurry and then stored at −80 °C. Cryo-sectioning was performed using a CM 1900 UV cryostat (Leica, Germany). Cochlear sections around the mid-modiolar plane were collected (12 µm; −22 °C) and mounted onto Superfrost-Plus slides (Menzel-Gläser, Braunschweig, Germany). Film and emulsion based autoradiography analyses were performed to assess drug distribution (Fig. 1).

### Autoradiography and radiolabel signal analysis

#### Quantitative standard establishment

Whatman filter paper disks with a diameter of 5 mm were used to establish a ^125^I-NT-3 concentration–gray intensity calibration curve. Ten 1:2 serial dilutions of ^125^I-NT-3 were prepared and 5 µL of each dilution was added with consistent strength to a filter paper disk. All disks with serial dilutions were arranged in straight rows on a strip of tape and fixed onto a cardboard with slides for film exposure.

#### Film-based signal visualization, re-visualization, and quantification

Radiolabel signal in different cochlear regions were visualized via film autoradiography and analyzed in MATLAB and ImageJ as described below.

Slides were thawed and air-dried. Together with the standards, the slides were taped onto a cardboard into a light-tight, radiation-shielded cassette with a Biomax MR film (Kodak) closely attached on top. The cassette was wrapped with foil and kept at −20 °C for 5–6 weeks to achieve adequate radiation exposure. Prior to exposure, the cassettes were brought back to room temperature for 30 min-1 h. The captured signal on film was then developed in a medical film processor (SRX-101A; Konica Minolta Medical and Graphic, Inc) and scanned with an Epson v800 photo scanner (grayscale, 16-bit, 2400 dpi with no compression). Post-exposure slides were stained with hematoxylin and eosin (H&E) and imaged for reference.

**Fig. 2:**
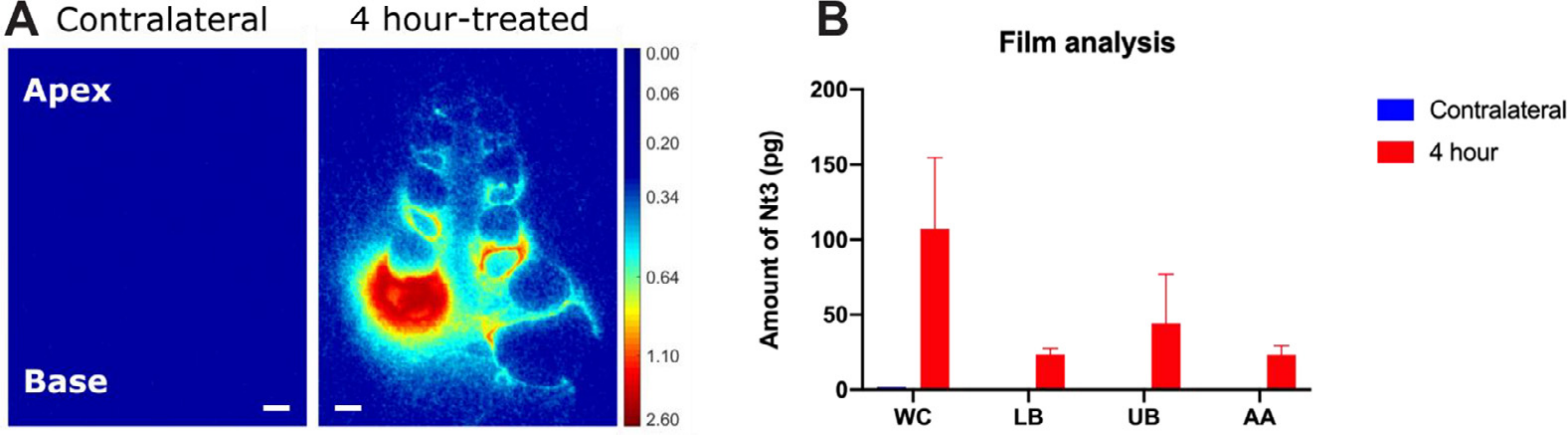
Autoradiography analysis on film using two 4 hour supraparticle-implanted cochleae as an example. (A) Representative color-mapped film radiographs of a supraparticle-implanted cochlea showing the widespread distribution of ^125^I-NT-3 across all cochlear regions and a base-to-apex declining concentration gradient at 4 hour post-treatment time point. In this example, the supraparticle was located in the upper basal turn of the cochlea. No radiolabel signal was detected in the contralateral untreated cochlea. Color bar indicates optical density from 0.00 to 2.60 with no normalization. Background was adjusted to be shown in dark blue. Color bar was not calibrated to show signal in pg due to varying standards across different pieces of film. Scale bar = 500 µm. (B) Quantification of amount of NT-3 at different regions of the cochlea after 4 h of intracochlear implantation treatment using the film analysis method. WC = whole cochlea; LB = lower basal turn; UB = upper basal turn; AA = all consecutive turns above basal turns. (For interpretation of the references to color in this figure legend, the reader is referred to the web version of this article.)

For each cochlea, a total of three cross-sections closest to the mid-modiolar plane (mid-modiolar sections, *n* = 3) were chosen from the digital radiographs based on the H&E sections. Selected sections were color-mapped via MATLAB R2019a to indicate region-wise signal distribution and distribution gradient (Fig. 2A). Supraparticle locations and possible tissue response were examined by inspecting all H&E sections available.

Region-based signal was analyzed in grayscale via Fiji version ImageJ software applying the developed calibration standards (Rodbard function for non-linear correlation fitting, r^2^ = 0.9915–0.9997). Standard region of interests (ROIs) were created for whole cochlea (WC), lower basal turn (LB), upper basal turn (UB), and all consecutive turns above basal turns (AA) using a polygon drawing tool (Fig. 1A). The standard ROIs were applied to the corresponding part of all the section samples with only minor spatial adjustments e.g. shape adjustment, rotation, and flipping to ensure a highly consistent area for signal measurement. A background (B) value was also measured using a standard ROI during each signal measurement. Area (mm^2^), mean gray value (pg per mm^2^), standard deviation, and integrated density (pg) were generated as outputs. Results were presented after background subtraction. For the result presented as integrated density (pg) (Fig. 2B), background and signal values were measured using the same ROI in order to factor in the same area (mm^2^) during value subtraction.

*Tips for film artefacts, detachment damage and foggy signal: 1. Watermarks can occasionally be seen in the film after development. That is possibly due to invasion of moisture from the environment during cassette transportation from the −20 °C freezer to the dark room (water can also damage the film by causing it to swell). To prevent watermark formation, cassettes can be placed into a vaccumed air-tight bag after collected from the freezer and also avoid putting the film onto the slides before they warm up to room temperature. 2. Removing the film from slides after exposure can sometimes damage the sections that are later stained with H&E for reference. To prevent this, we have tried dissolving the OCT on the slides in distilled water followed by 90% and 100% ethanol dehydration before exposure to film. From what we have observed, with this additional step implemented, all post-exposure sections have remained intact. 3. Foggy signal in the film where radioactive sections have been attached to suggests that film autoradiography requires extremely close distance between samples and the film. This can be improved by adding layers of cardboard into the cassette to reduce movable space.

#### Emulsion-based signal visualization, re-visualization, and quantification

Radio-sensitive emulsion was also used to visualize radiolabel signal in the cochlear tissue. Autoradiography and software-based signal analysis were performed as described below.

The slides were thawed and rinsed in water to remove the OCT. The slides were pre-stained with H&E following a standard protocol but doubling the time in dyes to overstain the samples. After that, both sets of slides were dehydrated in an escalating series of ethanol (70%, 90% and 100%) and air-dried. The sample side of the slides were coated with type NTB emulsion (Kodak) heated to 42 °C. Following coating, all slides were dried vertically in air for 5 min and transferred to a light-tight box for 1 h at room temperature. Following the addition of anhydrous silica, the slides were then transferred to 4 °C. At 5–6 weeks post-exposure, slides were brought back to room temperature for 1 h. Under safelight conditions, the slides were developed in 1:1 diluted Dektol developer (Kodak) in water for 5 min. Development was stopped by placing the slides in a water bath for 30 s and then in fixer (Kodak) for 10 min. The slides were washed in running water for 15 min, following which they were bathed successively in 90% ethanol, 100% ethanol, xylene, and then coverslipped with DPX.

**Fig. 3:**
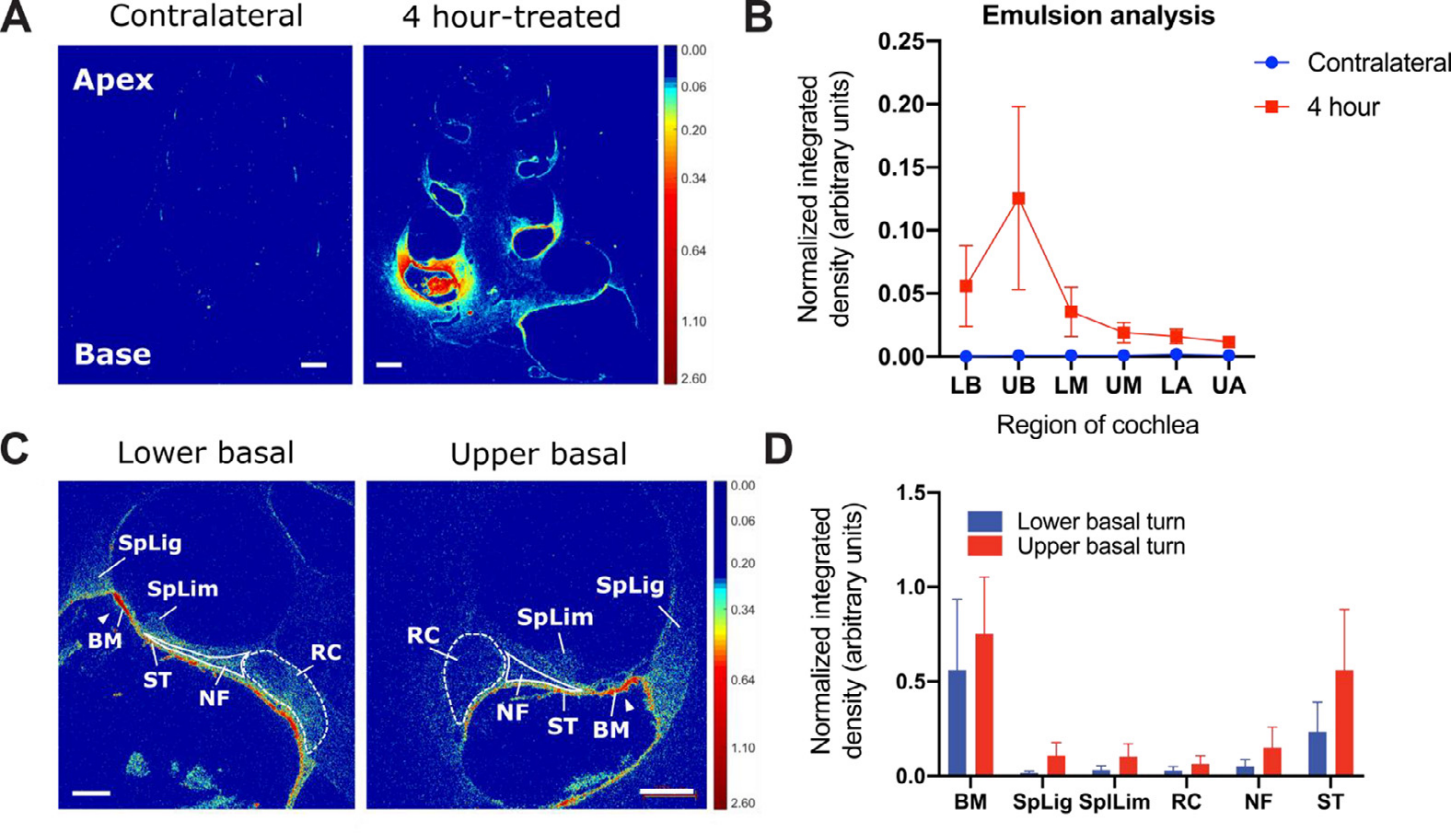
Autoradiography analysis on emulsion-coated sections using two 4 hour supraparticle-implanted cochleae as an example. (A) Representative color-mapped emulsion radiographs of a supraparticle-implanted cochlea showing widespread distribution of ^125^I-NT-3 across all cochlear regions and a base-to-apex declining concentration gradient at 4 h post-treatment time point. No radiolabel signal was asas detected in the contralateral untreated cochlea, although the pigmented cells from stria vascularis are visible. Color bar indicates optical density from 0.00 to 2.60 with no normalization. Background was adjusted to be shown in dark blue. Scale bar = 500 µm. (B) Quantification of ^125^I-NT-3 signal density at different regions of the cochlea after 4 h of intracochlear implantation treatment using the emulsion analysis method. LB = lower basal turn; UB = upper basal turn; LM = lower middle turn; UM = upper middle turn; LA = lower apical turn; UA = upper apical turn. (C) Higher magnification colormaps of lower and upper basal turns from the emulsion radiographs showing ^125^I-NT-3 uptake in the supraparticle-implanted cochlear tissue with highlights of uptake in the target tissue at 4 hour post-treatment time point. Dashed lines circle signal in the auditory nerve soma area; solid lines were drawn around signal in the auditory nerve fibers; solid triangles point at signal underneath the organ of Corti. Color bar indicates optical density from 0.00 to 2.60 with no normalization. Background was adjusted to be shown in dark blue. Scale bar = 200 µm. (D) Quantification of ^125^I-NT-3 signal density at different cochlear tissue in the lower and upper basal turns after 4 h of intracochlear implantation treatment using emulsion based analysis. BM = basilar membrane; SpLig = spiral ligament; SpLim = spiral limbus; RC = Rosenthal's canal; NF = nerve fibers; ST = mesothelial cell layer lining the scala tympani. (For interpretation of the references to color in this figure legend, the reader is referred to the web version of this article.)

For each cochlea, a total of four cross-sections closest to the mid-modiolar plane (mid-modiolar sections, *n* = 4) were selected for imaging via a Zeiss Axio Imager M2 upright microscope. The sections were imaged at 25X magnification to capture radiolabel signal across the whole cochlea using consistent microscope settings and acquisition settings. Image tiles were stitched together using the Zen software stitching function, allowing for 5% overlap. Lower and upper basal turns were imaged at 100X for a more detailed quantitative analysis on selected tissues. Signal in the target tissue (organ of Corti and Rosenthal's canal) of selected cochleae was captured at 200X as representatives. The imaged emulsion radiographs at 25X and 100X were color-mapped via MATLAB R2019a to indicate region-wise signal distribution, distribution gradient, and also tissue-based signal uptake (Fig. 3A and C).

Signal density quantification (densitometry) was performed in Fiji version ImageJ using images in grayscale. Calibration on measurement was set up based on the Kodak Calibrated No.3 Calibrated Step Tablet (available on https://imagej.nih.gov/ij/docs/examples/calibration/). Mean gray values for an 8-bit image (0–255) were converted to optical densities from 0 (white) to 3.05 (black). Rodbard function was used to fit a non-linear correlation (r^2^ = 0.9992). As depicted in Fig. 1B, ROIs were drawn carefully around 6 cochlear turns including lower basal turn (LB), upper basal turn (UB), lower middle turn (UM), upper middle turn (UM), lower apical turn (LA), and upper apical turn (UA) in each 25X image. A freehand tool was used, and the three fluid-filled chambers were excluded from all ROIs. In 100X basal-turn images, ROIs were carefully drawn around 6 types of tissue i.e. basilar membrane (BM) underneath the organ of Corti, spiral ligament (SpLig), spiral limbus (SpLim), Rosenthal's canal (RC) housing the auditory nerve soma, peripheral nerve fiber (NF), and the mesothelial cell layer lining the upper wall of scala tympani (ST). Background value was measured from a part of the cochlear section without visible signal. Area (mm^2^), mean gray value (optical density per mm^2^), standard deviation, and integrated density (optical density) were generated as outputs. Signal mean gray value (optical density per mm^2^ – arbitrary units) was used to present the emulsion densitometric results after background subtraction (Fig. 3B and D).

*Tips for emulsion artefacts: Emulsion-coated sections are sensitive to moisture during long-term exposure at 4 °C. We found it helpful to pre-stain the sections with H&E (standard protocol except for one quick dip in both dyes) prior to emulsion coating. Quantitative analysis can still be carried out after signal extraction (run ‘split channel’ on ImageJ and select ‘red channel’ for analysis). Using this method, although shades of H&E colors will no longer be distinguishable from slide background, it is still important to collect values from the tissue without signal for background subtraction.

The above-mentioned three techniques for visualizing and analyzing radiolabel dsitribution (film, emulsion-coated sections, and emulsion-coated sections stained with H&E counterstain) have advantages and disadvantages which can dictate the use of one over another. A summary of these strengths and weaknesses are provided in Supplementary Table 2.

## Conclusions

We present a novel method to assess drug pharmacokinetics in the cochlea using radiolabel techniques. We demonstrate that this method can be applied to obtain drug pharmacokinetics and biodistribution using whole cochlear gamma counts and autoradiography analyses of cochlear sections. Furthermore, it can be used to assess the spread of the drug to non-target tissue or the untreated cochlea using gamma counts as an indication of treatment safety. Apart from its application in the intracochlear drug delivery studies, this method is also readily adaptable to studies of other strategies delivering drugs to the cochlea, such as round window delivery. Here, it is important to note that not all drugs/substances can be radiolabeled and that post-labeling bioactivity testing is required prior to use. In our case, the radiolabeled NT-3 has been shown to remain bioactive *in vitro* primary auditory neuron survival assays [11,12]. Furthermore, the 59.4 day half-life of ^125^I needs to be taken into consideration for long-term pharmacokinetic experiments. It should also be taken into consideration that during the >2 week tissue processing times required for cochlear decalcification and cryopreservation low levels of NT-3 may continue to elute from the supraparticles and influence the distribution data. However, the protein cross-linking that occurs during fixation is expected to reduce this significantly. We expect this technique to not only be of interest to auditory researchers, but to be broadly applicable for pharmacokinetic studies in other systems.

## Declaration of Competing Interest

The authors declare that they have no known competing financial interests or personal relationships that could have appeared to influence the work reported in this paper.

## Appendix Supplementary materials

Image, application 1
