# Supplementary material for: A radiolabeled drug tracing method to study neurotrophin-3 retention and distribution in the cochlea after nano-based local delivery
Source: MethodsX. 2020 Sep 24;7:101078. doi: 10.1016/j.mex.2020.101078 (PMC7549113; doi:10.1016/j.mex.2020.101078)
Supplement: Supplementary file 1 [file mmc1.docx]

Supplementary Table 1 Comparison of techniques for inner ear pharmacokinetics.

| Techniques | Temporal resolution | Spatial resolution | Strength(s) | Weakness(es) |
| --- | --- | --- | --- | --- |
| Serial perilymph sampling | Depends upon sampling frequency | N/A | Method well-established;  Suitable to all types of drug;  Post-sampling drug concentration determination can be done with various measurement techniques based on laboratory equipment availability | PK limited to drug levels in the cochlear perilymph fluid;  Sampling volumes restricted by perilymph fluid available (less than 20 ul in small animals);  Possible contamination from cerebrospinal fluid depending on sampling techniques[6, 15];  Potential undesired binding of ‘sticky’ proteins to tissue during perilymph extraction |
| MRI/micro CT imaging[16, 17] | Real-time | Cochlear region level | Does not require animal termination;  Non-invasive during data collection | Unable to indicate uptake level of drug in the tissue;  Contrast agent behaviour might not resemble actual drug diffusion and clearance in the cochlea;  Requires special imaging equipment |
| Electrophysiological measurements[18] | Real-time | Cochlear region level (indicated by hearing frequency) | Data reflect *in vivo* drug effectiveness | Unable to determine delivered drug levels;  Measurements require acute drug response which limits application to a small range of drugs |
| Electrochemical measurements[6] | Real-time | Cochlear region level (achieved by multiple recording sites) | Allows determination of longitudinal drug flow rate | Unable to indicate uptake level of drug in the tissue;  Requires special recording equipment and an ionized drug |
| Fluorescent-labeled drug tracing[7, 19, 20] | Achieved via multiple animal individuals | Cellular level | Simple visualization via fluorescent microscopy | Tissue auto-fluorescence might affect data interpretation;  Large fluorophores might alter *in vivo* drug behaviour;  Photo-bleaching of fluorescent tags |
| Radiolabeled drug tracing | Achieved via multiple animal individuals | Cellular level | Duration of evaluation up to months depending on the radiolabels;  Allows for accurate determination of drug payloads in drug delivery systems, whole-cochlear drug levels and drug accumulation in off-target organs etc. in a relatively simple manner;  Maintains the integrity of the cochlea thereby enabling non-compromised analysis of cochlear distribution | Measurements need to account for natural radioactive decay;  Radioactive materials required;  Long exposure times required for visualization procedures |

Supplementary Table 2 Evaluation of techniques for radiolabeled drug distribution.

| Techniques | Strength(s) | Weakness(es) | Recommended use |
| --- | --- | --- | --- |
| Film | Easy to process with few artefacts;  Low labor requirement for processing of a large number of slides;  Easy to establish standards for drug quantification;  Signal amplified due to the nature of film material | Lacking resolution for accurate tissue-specific signal quantification | Use for signal/particle location inspection across a whole sequential series of sections (stain slides with H&E and scan for signal/particle location reference);  Whole-section/region-based drug amount evaluation on ImageJ;  Drug distribution map materials |
| Emulsion-coated sections | Cellular level resolution;  Suitable for accurate region of interests drawing;  More sensitive than film in visualizing low concentrations of drug | Relatively labor-intensive slide preparation and radiograph development;  Higher chance of artefacts;  Pigmented tissue and residual blood cells can confound the radiological signal | Use for region/tissue-specific signal quantification;  Drug distribution map materials |
| Emulsion-coated sections stained with H&E | Cellular level resolution with cells and tissue well visualized as reference | As per non-stained emulsion-coated sections | Use as research data representation and detailed signal/particle inspection;  Signal quantification can be performed (requires image processing to remove H&E stains) |

**References**

[15] A.N. Salt, C. Kellner, S. Hale, Contamination of perilymph sampled from the basal cochlear turn with cerebrospinal fluid, Hear. Res. 182 (1) (2003) 24–33.

[16] E.B. King, et al., Direct entry of gadolinium into the vestibule following intratympanic applications in Guinea pigs and the influence of cochlear implantation, J. Assoc. Res. Otolaryngol.: JARO 12 (6) (2011) 741–751.

[17] M. Haghpanahi, et al., Noninvasive technique for monitoring drug transport through the murine cochlea using microcomputed tomography, Ann. Biomed. Eng. 41 (10) (2013) 2130–2142.

[18] Z. Chen, et al., Inner ear drug delivery via a reciprocating perfusion system in the guinea pig, J. Control. Release: Off. J. Control. Release Soc. 110 (1) (2005) 1–19.

[19] W.S. Kang, et al., Intracochlear drug delivery through the oval window in fresh cadaveric human temporal bones, Otol. Neurotol.: Off. Publ. Am. Otological Soc. Am. Neurotol. Soc. Eur. Acad. Otol. Neurotol. 37 (3) (2016) 218–222.

[20] W.S. Kang, et al., Non-ototoxic local delivery of bisphosphonate to the mammalian cochlea, Otol. Neurotol.: Off. Publ. Am. Otological Soc. Am. Neurotol. Soc. Eur. Acad. Otol. Neurotol. 36 (6) (2015) 953–960.
